# Electromyography findings in L5 radiculopathy are not associated with fatty infiltration of paraspinal muscles: a retrospective observational study

**DOI:** 10.3389/fneur.2024.1447432

**Published:** 2024-12-16

**Authors:** Ekaterina Seliverstova, Mikhail Sinkin, Andrey Grin

**Affiliations:** N.V. Sklifosovsky Research Institute for Emergency Medicine, Moscow, Russia

**Keywords:** radiculopathy, electromyography, motor unit action potential (MUAP), disc herniation, paraspinal muscles, fatty infiltration

## Abstract

**Introduction:**

Increased fatty infiltration of the paraspinal muscles (PM) has been recognized as a sign of decreased muscle quality in patients with degenerative disc disease. However, whether fatty infiltration is a consequence of a neurogenic process due to spinal nerve root compression has not yet been determined.

**Objective:**

To investigate the correlation between fatty infiltration of the paraspinal muscles (PM) and neurogenic remodeling of motor unit action potentials (MUAPs) in patients with lumbar radiculopathy.

**Materials and methods:**

58 adult patients (32 women) with L5 radiculopathy caused by an L4-L5 herniated disc were examined. We compared the neurological status, EMG data of the PM at the L5 level on the affected side and the L4 level on the opposite side of the spine, and the severity of fatty infiltration of the PM assessed on axial T2-weighted magnetic resonance imaging (MRI) scans.

**Results:**

We did not find any correlation between the degree of fatty infiltration and neurogenic remodeling of MUAPs in the PM.

**Conclusion:**

The lack of correlation between the degree of fatty infiltration and the presence of neurogenic remodeling of MUAPs in the PM suggests that fatty infiltration and neuropathic changes in muscles may be considered as separate processes requiring further research.

## Introduction

The paraspinal muscles (PM) are composed of muscle groups adjacent to the vertebrae and are responsible for movement and stabilization of the spine. In the lumbar spine, these muscles include the multifidus, erector spinae, interspinales, intertransversarii, psoas major and quadratus lumborum (1). The multifidus muscles are important stabilizers of the lumbar spine (2).

The lumbar multifidus originates from the mammillary processes of the lumbar vertebrae and the posterior surface of the sacrum. It courses medially and cranially to insert on the spinous processes, two to five levels above their origin. Among the lumbar paraspinal muscles, the multifidus is the most crucial muscle as it contributes to almost two-thirds of spinal stability and is predominantly atrophied in patients with chronic low back pain (3).

Reduced range of motion in the spine, particularly in lumbar flexion, is associated with a high degree of fatty infiltration of the PM (4).

There are two hypotheses for the development of PM atrophy. The first one considers the atrophy to be caused by disuse, and the second one—by denervation due to spinal nerve root compression by a herniated intervertebral disc. In the latter case, the atrophic process is local (5). In patients with unilateral chronic radicular pain, the PM cross-sectional area is smaller on the symptomatic side (6). Moreover, histology reveals changes in the multifidus muscles at the level of the affected segment of the spine, which are caused by compression of the spinal nerve root. Histopathological data suggest a relatively low regenerative capacity of the multifidus muscles in patients with long-lasting radicular pain (7).

Electromyography (EMG) remains an important neurophysiological method for diagnosing radiculopathy and can be used to assess the functional state of compressed spinal nerve roots. EMG of the PM stands out as a way to identify electrographic signs of denervation and reinnervation processes in the deep muscles of the back (8). Denervation manifests as fibrillation potentials and positive sharp waves, whereas reinnervation can be observed as an increase in the amplitude, duration, and phases of MUAPs. Neuroanatomical and pathophysiological studies indicate that the innervation of the m. multifidus is very specific, whereas m. longissimus and m. iliocostalis may have multi-spinal nerve innervation.

The m. multifidus is innervated by the medial branch nerve of the posterior ramus of the spinal nerve at each level, which exits the spinal canal superolateral to the facet joint (1). Spinal nerve root compression leads to localized denervation changes in this particular muscle.

The purpose of this study was to determine the relationship between PM neuropathic changes measured by EMG and the degree of fatty infiltration on the affected and unaffected sides.

## Materials and methods

A retrospective observational study was performed in the Clinical Neurophysiology Laboratory of the Emergency Neurosurgery Department, N.V. Sklifosovsky Research Institute for Emergency Medicine.

### Inclusion criteria

Clinical manifestations of unilateral radiculopathy L5 (radicular pain syndrome (leg pain), motor deficit in the form of weakness of the extensor muscles of the foot and/or big toe, sensory loss in the L5 dermatome, decrease/loss of the medial hamstring tendon reflex) caused by L4-L5 intervertebral disc herniation, confirmed by MRI. Disease duration was from one to 12 months after onset.

### Exclusion criteria

Patients with severe pain (VAS score of 9 to 10), disease duration of less than 4 weeks, history of spinal surgery at any segment, anatomical variants of the spine (S1 lumbarization, L5 sacralization), and neuromuscular disease were not included in the study.

### The study design

The study design covered standard neurological examination, radicular pain localization and intensity evaluation (VAS score), estimation of motor deficit according to the Medical Research Council Weakness Scale (MRC), and sensory loss assessment.

The participants with clinical manifistation of L5 radiculopathy were consecutively selected in order of hospitalization in neurosurgery department (consecutive sampling). All patients were to undergo spinal surgery. The sampling process came to an end when the time limit (1 year) was reached.

### Сlinical assessment

The clinical assessment in the study involved the following key points:

Patient Characteristics: The study included patients with unilateral L5 radiculopathy, characterized by radicular pain, motor deficits, and sensory loss as confirmed by clinical evaluations and MRI.

Neurological Examination: Each patient underwent a standard neurological examination, which included evaluation of radicular pain localization and intensity using a Visual Analog Scale (VAS), as well as assessment of motor deficits according to the Medical Research Council Weakness Scale (MRC).

Grouping by Disease Duration: Patients were stratified based on the duration of symptoms prior to EMG testing. The groups are as follows:

1. Less than 3 months: Patients in this group had a disease duration of fewer than 3 months before EMG testing.
2. Four to 6 months: This group included patients whose symptoms had persisted for between 4 to 6 months.
3. More than 6 months: Patients in this group had been experiencing symptoms for more than 6 months prior to the EMG.

This division was made to analyze the correlation between the duration of the disease and the electrodiagnostic and MRI findings related to the paraspinal muscles.

### EMG assessment

EMG was performed via *Skybox* equipment (*Neurosoft*, Russian Federation). А coaxial bipolar electrodes (*l* = 75 mm, *d* = 0.6 mm, *Neurosoft*, Russian Federation) were used. The protocol included the examination of the multifidus muscles at the L5 level on the affected side and L4 level on the opposite side to evaluate the neurophysiological differences. Specifically:

Affected Side (L5): The focus was on the L5 level on the affected side to assess the impact of spinal nerve root compression. This allowed for evaluation of motor unit action potentials (MUAPs) to identify neuropathic changes associated with the nerve injury.

Opposite Side (L4): The L4 level on the opposite side served as a control to observe how the muscle’s electrical activity was affected by the unilateral radiculopathy. This comparison offered insight into how the neuromuscular function might differ in regions not directly affected by nerve compression, thus providing a baseline for understanding the changes that occur on the symptomatic side.

**Figure 1:**
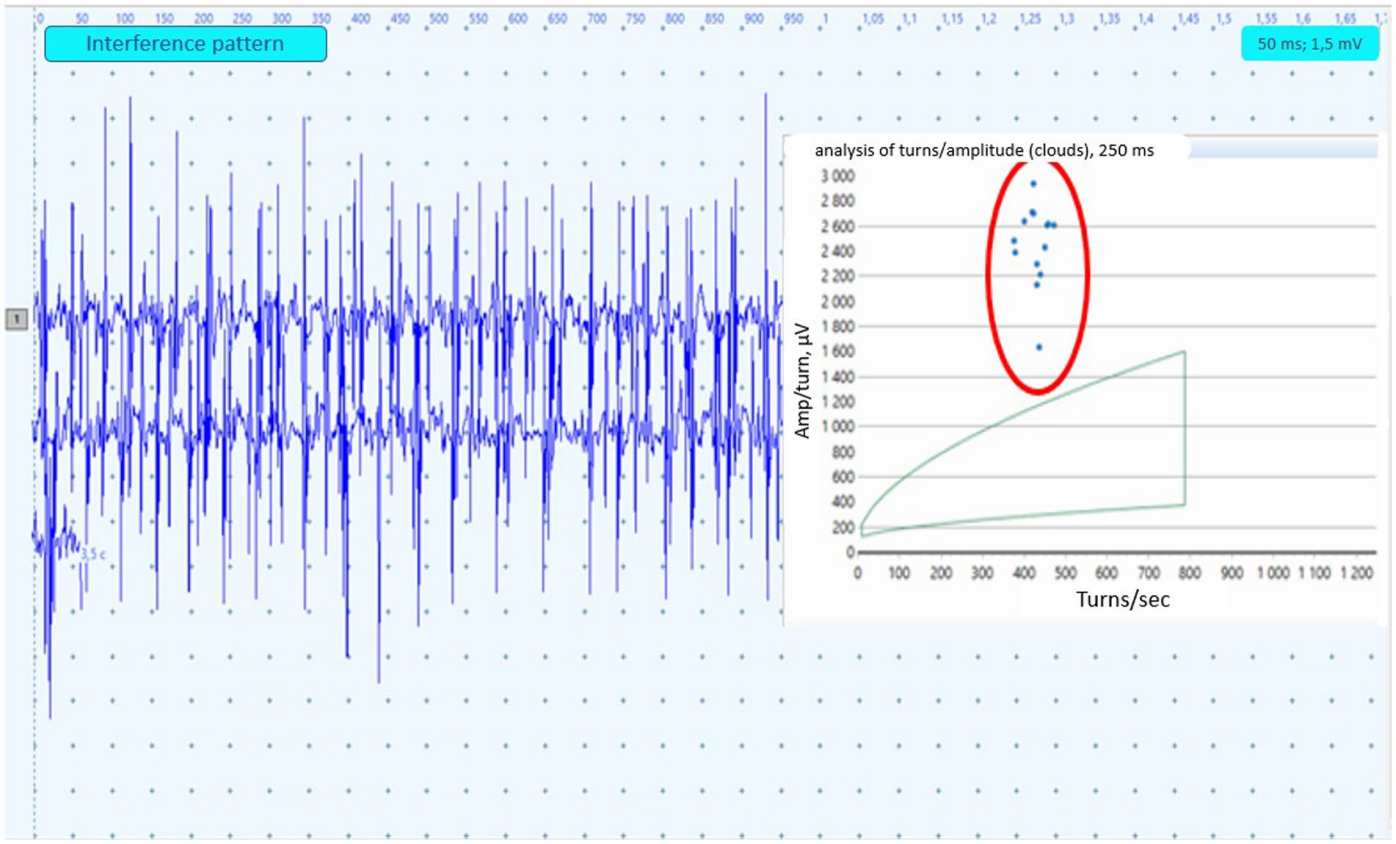
Example of the EMG neuropathic interference pattern in paraspinal muscles. The turns-amplitude analysis cloud corresponds to 95% confidence age-matched limits. Dots circled in red represent the result of PM analysis from 250 msec. Epochs.

A sample of 20 MUAPs were extracted. MUAP duration was defined as the time between the start and end points of the MUAP when observed at a sensitivity of 100 μV/cm and a sweep screen of 10 ms/cm. We did not use the software-based assessment of MUAPs and made changes such as excluding inappropriate software-selected MUAPs. The mean and maximum amplitude and duration of MUAPs, the number of phases and interference pattern analysis (quantitative and qualitative) were investigated (Figure 1). An increase in mean MUAPs duration combined with a reduced interference pattern were classified as neurogenic findings. At least three different sites within each muscle were sampled and analyzed for MUAP morphology and recruitment.

The patient was in the prone position throughout the study. Muscle activation was achieved by lifting the homologous leg. The skin penetration point was located 2.5 cm lateral to the spinous process of the L4 and L5 vertebrae, correspondingly. The coaxial bipolar electrode was inserted at a 45-degree angle to the skin surface.

The reference values used for MUAPs analysis (amplitude and duration) are presented in Table 1.

**Table 1:** Mean MUAPs amplitude and duration in m. multifidus at L5 level in healthy people according to Marco Tomasella (10).

Parameter	M ± SD
Duration (ms)	11.6 ± 2.2
Amplitude (μV)	723 ± 167

### MRI assessment

**Figure 2:**
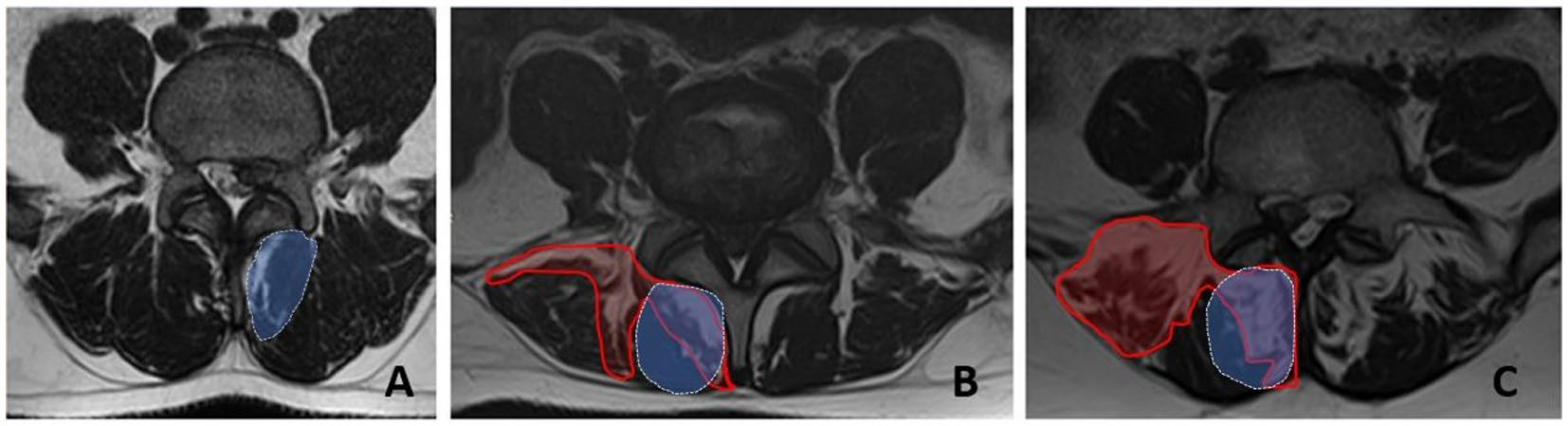
Fatty infiltration grades for the paraspinal muscles at the L5 level measured by MRI (axial sections). **A** - grade 0, **B** - grade 1, **C** - grade 2. The area of fatty infiltration is circled in red. The area of m.multifidus is circled in blue.

Visual assessment of the PM structure was based on axial T2-weighted MRI sections at the level of the herniated L4-L5 disc. The severity of PM fatty infiltration was used to divide the patients into 3 groups: “Grade 0” - 0-10%, “Grade 1” - 10-50%, and “Grade 2” - more than 50%. In general, the degree of fatty infiltration of PM was previously reported to be correlated with that in isolated m. multifidus (Figure 2) (9).

### Statistical data processing

Statistical data processing was performed using the *SPSS* 26 application package. Median, 25th and 75th percentiles were calculated for participants’ characteristics. The nonparametric Wilcoxon test (for pairwise comparisons) and the Friedman test (for multiple comparisons) were used to compare electromyography parameters. Chi square test was used to evaluate the differences between the groups. The regression equations linking the EMG parameters of PM (mean amplitude and duration of MUAPs at the L5 level on the side of spinal nerve root compression) with the degree of fatty infiltration of PM were estimated.

Differences were considered statistically significant at *p* < 0.05.

## Results

The study cohort comprised 58 patients aged between 26 and 73 years, with a median age of 44 years (interquartile range: 41–54). Among these participants, 32 (55.2%) were female (Table 2). Radicular pain of varying severity was observed in all patients. Furthermore, motor deficits were present in 51.7% of the patients. Numbness in the L5 dermatome was reported in 26 patients (44.8%). The duration of disease before spinal surgery ranged from four to 48 weeks (Me 12, Q1-Q3:4–16).

**Table 2:** Patients’ characteristics (*n* = 58).

Patient characteristics	Me, Q1–Q3 or %
Age	44, 41–54
Gender (female)	55, 2%
Leg pain duration (weeks)	12, 4–16
Right	41.5
Left	58.5
Leg pain score (VAS)	6.5, 5–8
Fatty infiltration	
Grade 0	31
Grade 1	45
Grade 2	24
Neuropathic MUAPs	82,8

Me, Median. Q1–Q3, interquartile range. VAS, Visual Analog Scale. MUAPs, motor unit action potentials.

The mean and maximal MUAPs amplitude and duration at the L5 level on the affected side and L4 level on the opposite side are presented in Table 3.

**Table 3:** Comparison of the mean and maximal MUAPs amplitude and duration of the paraspinal muscle at L5 level on the affected side and L4 level on the opposite side.

	Mean Amp. (μV) Me, Q1–Q3	Max Amp. (μV) Me, Q1–Q3	Mean Dur.(ms) Me, Q1–Q3	Max Dur (ms) Me, Q1–Q3
L5 affected side	1,486 (1351–1916)	3,276 (2405–3,933)	11,9, (11,2-12,4)	16,3 (14,5-17,9)
L4 opposite side	1,206 (990–1,660)	2,135 (1860–3,227)	10,9 (10,7-11,9)	14,8 (12,3-15,9)

Amp., amplitude. Dur., duration. Me, Median. Q1–Q3, interquartile range.

**Figure 3:**
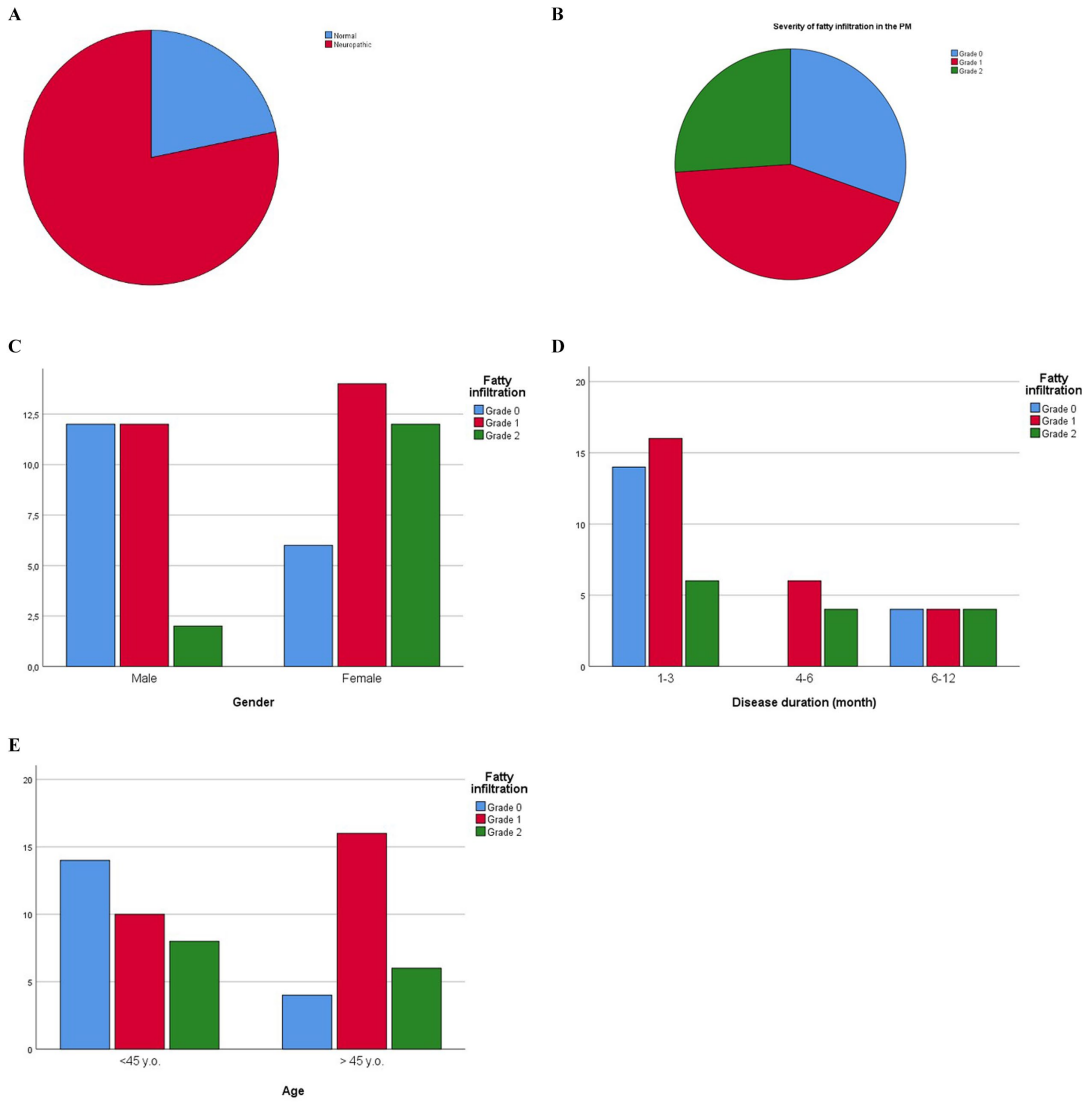
**(A)** Distribution of patients with neurogenic remodeling of MUAPs in the paraspinal muscles. Pie chart representing the percentage of normal (blue) and neuroparhic (red) EMG pattern is shown. **(B)** Distribution of patients depending on the degree of PM fatty infiltration (FI). Pie chart representing the percentage of patients with Grade 0 (blue), Grade 1 (red) or Grade 2 (green) FI is shown. **(C)** Different degrees of PM fatty degeneration in male and female patients. Bar plots representing 26 of male (left) and 32 female (right) patients with different FI grades are shown. **(D)** Distribution of patients with different degrees of PM fatty infiltration and disease duration. Bar plots representing patients with different FI grades depending on the disease duration are shown. **(E)** Distribution of patients with different degrees of PM fatty infiltration and age. Bar plots representing patients with different FI grades depending on patient’s age are shown.

No statistically significant differences in the mean and maximum MUAPs amplitude at the L4 and L5 levels on the symptomatic and contralateral sides were found (*p* > 0.05). The mean and maximum duration of MUAPs at the L5 level on the affected side were statistically significantly different from of MUAPs at the L4 level on the opposite side (*p* < 0.001). The mean duration of MUAPs on the contralateral side corresponded to the normative values calculated by Marco Tomasella (10). On the symptomatic side they were abnormal and a total of 48 (82.8%) patients demonstrated a neuropathic pattern in the PM (Figure 3A).

The degree of fatty infiltration was almost identical on the symptomatic and non-symptomatic sides. The PM structure did not change in 18 (31%) patients. The severity of fatty infiltration corresponded to Grade 1 in 26 (45%) patients and to Grade 2 in 14 (24%) patients (Figure 3B). Fatty infiltration (FI) in the PM was significantly more often observed in females (*p* < 0.05; Figure 3C). The presence of FI in the PM did not depend on the disease duration (*p* > 0.05; Figure 3D). In patients over 45 years old, fatty infiltration of the PM was statistically more frequent (*p* < 0.05; Figure 3E).

**Figure 4:**
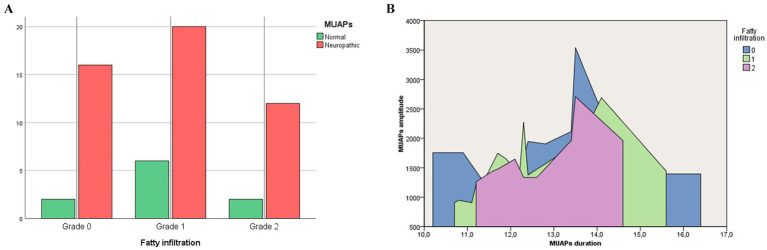
Rate of neurogenic remodeling of MUAPs **(A)** and the average amplitude (mV) and duration (ms) of MUAPs **(B)** in patients with different degrees of fatty infiltration.

The rate of neurogenic remodeling of MUAPs was equal in patients with different degrees of FI (*p* > 0.05; Figure 4).

Correlation analysis did not reveal any relationship between the severity of fatty infiltration and the presence of neurogenic remodeling of MUAPs in the PM (Table 4).

**Table 4:** The results of assessing regression equations relating PM EMG parameters (the mean amplitude and duration of MUAPs at the L5 level on the affected side and at the L4 level on the unaffected side) to the degree of PM fatty infiltration.

Indicators	Coefficient	*t*-statistic	*р*	Elasticity coefficient	R^2^	F-statistic	*р*
Average duration of MUAPs (L5)							
*Grade 1*	0	−0.01	0.991	−0.50%	0.06	1.91	**0.158**
*Grade 2*	−0.75	−1.69	0.097	−5.80%			
*Constant (grade 0)*	13.49	46.17	<0.001	–			
Average amplitude of MUAPs (L5)							
*Grade 1*	−261.89	−1.5	0.141	−13.10%	0.04	1.12	**0.334**
*Grade 2*	−157.6	−0.77	0.442	−5.70%			
*constant (grade 0)*	1843.89	13.7	<0.001	–			
*Grade 2*	167.51	0.36	0.724	8.80%			
*constant (grade 0)*	2437.78	7.82	<0.001	–			
Average duration of MUAPs (L4)							
*Grade 1*	−0.28	−0.48	0.63	−1,5%	0.01	0.28	**0.756**
*Grade 2*	0.16	0,25	0.806	2,5%			
*Constant (grade 0)*	12.22	27,90	<0,001	–			
Average amplitude of MUAPs (L4)							
*Grade 1*	48.8	0.29	0.776	1.40%	0.04	1.14	**0.326**
*Grade 2*	282.4	1.42	0.16	20.80%			
*Constant (grade 0)*	1281.89	9.78	<0,001	–			

*coefficients are significant at the 5% level, ** at the 1% level. MUAPs, motor unit action potentials. Bold values in the table indicate results that are not statistically significant, where *p*-values are greater than 0.05 (*p* > 0.05).

## Discussion

Recent publications have described fatty infiltration of the PM as a natural ageing process (11). The development of muscle disuse atrophy is also promoted by a sedentary lifestyle. Muscles undergo adaptive remodeling in response to physical inactivity and aging (12). Several studies have shown that PM fatty infiltration can be detected both in patients with acute or chronic low back pain and healthy individuals and, therefore it is not a pain-specific feature.

Current data on the factors influencing the development of PM fatty infiltration is contradictory. For example, M. Hildebrandt et al. reported that PM fatty infiltration is not dependent on the duration of the back pain, patient’s age, sex, and body mass index (BMI) (4). On the other hand, a study by X. Peng et al. showed that PM fatty infiltration increases with age and BMI (13). According to another study by V. G. Felipe et al., the area and severity of PM fatty infiltration does not correlate with age and is more often observed in women (14).

There are two ways for adipocyte accumulation in skeletal muscles (15). First, lipids can be accumulated within myofibrils (intramyocellular lipids) and such accumulation is associated with insulin resistance, inflammation, and lack of muscle functional activity (16).

The second mechanism of myosteatosis is associated with intermuscular fat accumulation. There are several populations of stem cells in skeletal muscles, the most well-defined of which are muscle satellite cells, which lie beneath the basal lamina of muscle fibers and contribute to myogenesis during muscle regeneration. Another cell population, which was recently described, is called fibroadipogenic progenitors or mesenchymal interstitial cells, which readily differentiate into adipocytes under various conditions such as muscle injury or glucocorticoid treatment (17). Endogenous glucocorticoid levels increase with age, which may contribute to intermuscular fat deposition (18). Moreover, anxiety and depressive disorders are associated with the hypersecretion of corticotropin-releasing hormone, which leads to high levels of circulating endogenous glucocorticoids (19).

Estrogen deficiency increases skeletal muscle lipid content and adipogenic gene expression and decreases the relative share of satellite cells in ovariectomized rodents (20). In contrast, androgen deprivation therapy also increases fatty skeletal muscle infiltration in men with prostate cancer, although computed tomography does not distinguish between intramyocellular and intermuscular lipid accumulation; therefore, the actual location of lipid deposition is unclear in this case (21). Taken together, these data indicate that many conditions that induce bone marrow adipogenesis and bone loss in men and women, such as disuse atrophy, sex hormone deficiency, and changes in endogenous glucocorticoid levels, also stimulate the accumulation of adipocytes and intramyocellular lipids in skeletal muscles.

The available data may serve as a basis for further research into the correlation between hormonal status and the severity of fatty infiltration, as well as for clarifying the mechanism of myosteatosis to identify potential therapeutic strategies and prevention.

We hypothesized that the cause of fatty infiltration of the PM could be neurogenic changes due to spinal nerve root compression. After denervation, muscle passes through immediate loss of voluntary function and rapid loss of mass, increasing atrophy and muscle fiber degeneration and replacement of muscle by fibrous connective tissue and fat. In the PM, specifically in the multifidus muscles, we have identified neurogenic changes of MUAPs on the side of L5 radiculopathy. In contrast, the MUAPs parameters on the unaffected side were within normal limits, regardless of the degree of fatty infiltration. These findings allowed us to conclude that the processes of neurogenic remodeling of MUAPs in multifidus muscles and their fatty infiltration are independent.

Our study indicated that the frequency of fatty infiltration in PM was higher in females, potentially due to hormonal differences, such as estrogen deficiency, which can influence muscle lipid content.

The findings lend support to the hypothesis that more research is needed to explore the mechanisms behind fatty infiltration, to better understand their implications for treatment and prevention strategies in clinical settings.

### Limitation of the study

There may be some possible limitations in this study.

First, patients experiencing severe pain (VAS score of 9 to 10), were excluded (due to EMG intolerance), which could lead to a selection bias and limit the applicability of the results to individuals with more moderate pain levels. Additionally, participants with a disease duration of less than 4 weeks were also excluded, potentially omitting data on early-stage patients. Furthermore, the inclusion of participants who underwent spinal surgery may limit the generalizability of the findings, as the surgical context may not reflect the experiences of patients with similar conditions who have not undergone such interventions. These limitations highlight the necessity for further research to validate the findings across a more diverse population and to incorporate longitudinal assessments that capture long-term outcomes.

## Conclusion

There is an absence of correlation between the degree of fatty infiltration and the presence of neuropathic EMG findings. Thus, we should probably consider fatty infiltration and neurogenic changes in the muscles as two independent processes both requiring further in-depth research.

## Data Availability

The raw data supporting the conclusions of this article will be made available by the authors, without undue reservation.
